# Islets and Glucose Homeostasis

**DOI:** 10.1155/2015/358460

**Published:** 2015-03-03

**Authors:** Tien-Jyun Chang, Gang Xu, Jyuhn-Huarng Juang

**Affiliations:** ^1^Department of Internal Medicine, National Taiwan University Hospital, Taipei 10002, Taiwan; ^2^Department of Medicine and Therapeutics, The Chinese University of Hong Kong, Shatin, Hong Kong; ^3^Division of Endocrinology and Metabolism, Department of Internal Medicine, Chang Gung University and Chang Gung Memorial Hospital, Taoyuan City 33305, Taiwan

Diabetes mellitus is a chronic progressive metabolic disease, resulting from both insulin resistance and the dysfunction of beta-cells. Beta-cell apoptosis is a crucial pathophysiology leading to diabetes [1]. Aberrant immune system leads to destroying of beta-cells occurring in type 1 diabetes. Infiltration of immune cells around beta-cells and attack of beta-cells by cytokines or chemokines through upregulating the proapoptotic molecule Bid and subsequently the release of cytochrome c from mitochondria contributed to apoptosis. Fas/FasL and TNF pathways also elicit the same downstream molecules as the above-mentioned apoptotic pathway [2]. Unlike type 1 diabetes, metabolic disorders mainly cause type 2 diabetes, such as chronic glucotoxicity, lipotoxicity, and endoplasmic reticulum (ER) stress [3, 4]. Apoptosis in pancreatic beta-cells is observed in response to various stimuli such as glucose, cytokines, free fatty acids, leptin, islet amyloid polypeptide, ER stress, and sulfonylureas [4, 5]. Regardless of the underlying cause of the disease, insufficient beta-cell mass leads to dependence on exogenous insulin for blood glucose regulation. The morbidities associated with diabetes are significant. The knowledge about pancreas or islets transplantation and factors attributing to changing the secretory function and/or mass of islet beta-cells might help to develop a novel treatment to diabetes.

The past 15–20 years has seen a dramatic increase in the prevalence of T2D in children and adolescents [6–8]. Type 2 diabetes is generally believed to be a polygenic disorder, with disease development being influenced by both hereditary and environmental factors [9]. Support for the role of genetic factors comes from epidemiological evidence that type 2 diabetes in youth is most common in individuals from racial groups with a high prevalence of diabetes and in individuals with a strong family history [10]. A search for the contribution of certain candidate genes in the early onset type 2 diabetes is mandatory for further understanding of pathogenesis of type 2 diabetes in childhood. In this issue, Y.-D. Jiang et al. reported that E23K polymorphism of the* KCNJ11* gene contributed to an increased risk for type 2 diabetes in school-aged child and adolescence. K23-allele-containing genotypes conferring increased plasma insulin level during OGTT in normal subjects. However, the diabetic subjects with the K23-allele-containing genotypes had lower fasting plasma insulin levels after adjustment of age and BMI percentiles.

T2DM is a multifactorial metabolic disease mainly characterized by hyperglycemia [11], but before the occurrence of overt hyperglycemia, peripheral insulin resistance leads to compensatory insulin hypersecretion by pancreatic islets [12]. A. P. O. Protzek et al. reported that augmented *β*-cell function and increased *β*-cell mass developed in response to the glucocorticoid-induced insulin resistance involve inhibition of the islet AS160 protein activity.

Recently, human islet transplantation has achieved insulin independence in type 1 diabetes and the success rates have been markedly improved [13]. However, most successful cases need 2 or more implants and the long-term follow-up shows their insulin independence declines with time [14, 15]. Therefore, the critical issue in clinical islet transplantation is to further improve and maintain its successful rate. J.-H. Juang et al. reported that posttransplant DPP-4 inhibition with MK-0431 in the diabetic recipient with a marginal number of islets is not beneficial to transplantation outcome or islet grafts. We cannot exclude the possibility that higher dose of MK-0431 or more islets graft may have beneficial effects on the outcome of islet transplantation. M. A. Kanak et al. reviewed recent advances in understanding the role of inflammation in islet transplantation and development of strategies to prevent damage to islets from inflammation. Details on cell signaling pathways in islets triggered by cytokines and harmful inflammatory events during pancreas procurement, pancreas preservation, islet isolation, and islet infusion are presented. The authors also discussed several potent anti-inflammatory strategies that show promise for improving islet engraftment.

Type 1 diabetes is characterized by the progressive loss of pancreatic beta-cells caused by autoimmune attack [16]. Although beta-cell mass is markedly diminished in long-standing type 1 diabetics, residual beta-cells can be detected and new beta-cell formation may occur in these patients several decades after the disease onset [17]. This observation has led to researches to induce remission of diabetes by targeting beta-cell autoimmunity and regeneration. J.-H. Juang et al. reported that before the onset of autoimmune diabetes, all-*trans*-retinoid acid (ATRA) and exendin-4 treatment alone preserves pancreatic beta-cells; ATRA and ATRA plus exendin-4 treatment delays the onset of autoimmune diabetes. However, after the onset of autoimmune diabetes, ATRA and/or exendin-4 treatment is unable to reverse hyperglycemia or improve survival.

*Tien-Jyun Chang* *Tien-Jyun Chang* *Gang Xu* *Gang Xu* *Jyuhn-Huarng Juang* *Jyuhn-Huarng Juang*
